# Use of viability PCR for detection of live Chlamydia trachomatis in clinical specimens

**DOI:** 10.3389/frph.2023.1199740

**Published:** 2023-08-04

**Authors:** Lucia Vojtech, Shahrokh Paktinat, Tiffany Luu, Stella Teichmann, Olusegun O. Soge, Robert Suchland, Lindley A. Barbee, Christine M. Khosropour

**Affiliations:** ^1^ Department of Obstetrics and Gynecology, University of Washington, Seattle, WA, United States; ^2^ Department of Medicine, University of Washington, Seattle, WA, United States; ^3^ Department of Global Health, University of Washington, Seattle, WA, United States; ^4^ Public Health – Seattle and King County, HIV/STD Program, Seattle, WA, United States; ^5^ Department of Epidemiology, University of Washington, Seattle, WA, United States

**Keywords:** Chlamydia, PCR, sexually transmitted infection, nucleic acids, NAAT, DNA extraction

## Abstract

**Background:**

The current testing approach to diagnose *Chlamydia trachomatis* (CT) infection relies on nucleic acid amplification tests (NAATs). These tests are highly sensitive, but do not distinguish between active infection and residual bacterial nucleic acid which may remain after resolution of infection, or via cross-contamination. Better methods to assess the viability of CT detected in clinical samples would be useful in determining the relevance of CT detection in a variety of clinical settings. The goal of this study was to test viability PCR (vPCR) as a method to distinguish viable bacteria from non-viable CT.

**Methods:**

The vPCR relies on a propidium monoazide dye (PMAxx), which intercalates into accessible DNA from dead organisms and prevents their detection in a PCR assay for the CT *ompA* gene. We used digital PCR to quantify absolute genome copy numbers from samples. We validated the vPCR approach using laboratory stocks of CT with known viability. Then, we tested total DNA, viable CT DNA, and culture results from 18 clinical vaginal specimens and 25 rectal clinical specimens, all of which had tested positive by NAAT.

**Results:**

In laboratory stocks of CT, vPCR using defined ratios of heat-killed to live bacteria tracked closely with expected results. In vaginal clinical specimens, vPCR and total DNA results were correlated, though total DNA genomes outnumbered viable genomes by 2.2–52.6-fold more copies. As expected, vPCR detected more total genomes than culture results. Both vPCR and total DNA correlated with culture results (Spearman correlation *R* = 0.8425 for total DNA and 0.8056 for vPCR). Ten rectal NAAT positive specimens were negative by total DNA PCR, vPCR, and were negative or inconclusive by culture. Of the 6 rectal specimens that were culture positive, all were total DNA and vPCR positive. vPCR additionally detected viable bacterial DNA in 8 specimens which were NAAT + and culture negative, though levels were very low (mean 1,357 copies/ml)

**Conclusions:**

vPCR is a fast and easy method to assess viability in clinical specimens and is more correlated with culture results than total DNA PCR. Inconsistent ratios between total DNA and vPCR results suggest that the amount of dead bacteria varies considerably in clinical specimens. Results from rectal specimens suggest that many NAAT positive specimens do not in fact represent live replicating bacteria, and likely result in significant overuse of unnecessary antibiotics.

## 1. Introduction

Chlamydial infections caused by *Chlamydia trachomatis* (CT) serovars D-K are the most common bacterial sexually transmitted infections (STIs) worldwide (1). CT is typically asymptomatic but can lead to adverse health outcomes. CT genital infection causes pelvic inflammatory disease, ectopic pregnancy and tubal factor infertility (2). Chlamydial infection of the lower gastrointestinal tract (known as “rectal CT”) may lead to proctitis and can increase the risk of HIV acquisition and transmission (3–5). Due to its asymptomatic nature, many individuals infected with CT are never diagnosed, which may increase the risk of sequelae and lead to transmission to sexual partners (6).

The current diagnostic gold standard for CT is nucleic acid amplification testing (NAAT). Various NAAT platforms detect bacterial ribosomal RNA or DNA typically with amplification of nucleic acids, dramatically increasing sensitivity. However, NAATs are not able to distinguish between active (“true”) infections with viable, replicating bacteria that have infected cells, vs. detecting cell-free nucleic acid or nonviable bacteria. Furthermore, because NAAT targets occur in variable copy number per organism they cannot be used to determine bacterial load (7). Thus, it is possible that a sizeable proportion of NAAT-positive tests detected clinically may not actually represent active infections. Provision of antibiotic treatment for these “infections” is unnecessary and may lead to subsequent antibacterial resistance.

CT culture remains the gold standard to demonstrate replication competent infection, but culture often has low sensitivity and is difficult to implement, in particular with rectal samples, making it less preferable for diagnostic testing relative to NAAT. Developing a rapid, easy-to-implement assay that could detect viable CT would substantially advance the field by offering a method to identify infections that should be treated (i.e., those with viable CT) vs. those that do not require treatment (i.e., those with only CT nucleic acid but not live replicating bacteria).

CT viability assays have been employed to demonstrate that trachoma-causing strains of CT survive on surfaces and may contribute to the transmission of CT to the eyes (8). Viability assays have also been used in genital tract samples by Janssen and colleagues, who reported that about one quarter of vaginal swabs and about half of rectal swabs positive by NAAT did not contain viable CT DNA (9, 10). This group has also used viability PCR to assess bacterial load and spontaneous clearance (11, 12). However, their analyses have not compared viability assay to culture results for clinical specimens, so it remains unclear how well a viability assay performs against culture, the gold standard for detecting viable bacteria, in vaginal or rectal swabs.

In this study we analyzed a viability PCR (vPCR) assay to examine the ability of the assay to distinguish live from dead bacteria in clinical samples, and to assess whether vPCR is a reasonable alternative to CT culture for confirmation of active infection in clinical specimens. Specifically, we compared vPCR, total DNA, and culture results from vaginal and rectal NAAT + clinical specimens. We propose vPCR as a valuable tool in stratifying NAAT + results as likely to be active infections or resulting from low level or transient bacterial DNA contamination.

## 2. Methods

### 2.1. Generation of CT stock

Strains used for this study were propagated from a collection of frozen samples initially collected from women attending the Seattle King County Health Department STD Clinics from 1986 through 2010 and stored at the University of Washington Chlamydia Repository (13). Specimen collection, culture isolation techniques, and serotyping were conducted as described previously (14). Briefly, patient swabs were collected and stored in *Chlamydia* transport medium at 4°C and were transported to the laboratory within 24 h. Each specimen was inoculated onto McCoy cells, centrifuged at 1,200 × *g*, aspirated, and overlaid with minimal essential medium to which 10% fetal bovine serum and cycloheximide had been added (MEM-10). Cells were incubated at 37°C under 4% CO_2_ for 48 h and then passaged to increase titer. Chlamydial growth was detected by fluorescence microscopy using the genus-specific monoclonal antibody EVI-H1 (a gift from Harlan Caldwell). Recovered archival isolates were then cloned by a twofold limiting dilution method. The resulting cloned elementary bodies (EBs) were grown to high titers and were partially purified by centrifugation of lysates of infected cells through a 30% MD-Gastroview® pad (Mallinckrodt Inc. St Louis).

### 2.2. Culturing assay

Culture assays were repeated on the same 1x frozen and thawed aliquots used for vPCR and total DNA results. Samples were thawed, diluted, and inoculated onto McCoy cells in duplicated wells, centrifuged at 1,200 × *g*, aspirated, and overlaid with minimal essential medium to which 10% fetal bovine serum and cycloheximide had been added (MEM-10). Cells were incubated at 37°C under 4% CO_2_ for 48 h and were fixed with methanol. Chlamydial growth was detected by fluorescence microscopy using the genus-specific monoclonal antibody EVI-H1 (a gift from Harlan Caldwell).

### 2.3. Clinical specimen collection

Vaginal specimens and the resulting chlamydial cultures used in this study were initially collected as part of routine clinical care from women presenting to the Seattle King County Health Department STD Clinics from 1986 through 2010 and stored frozen in the University of Washington Chlamydia Repository (1). Specimen collection, culture isolation techniques, and serotyping were conducted as described previously (2). Briefly, patient swabs were collected and stored in Chlamydia transport medium at 4°C and were transported to the laboratory within 24 h.

Rectal specimens were collected in 2019–2022 as part of the “Bottom's Up” study, which has been previously described (15). Briefly, recruitment took place at the municipal sexual health clinic in Seattle, Washington. Individuals who were assigned male sex at birth, at least 16 years old, reported sex with men in the last year, but no receptive anal sex in the past 2 years were eligible to participate. These eligibility criteria reflect the objectives of the Bottom's Up study, which was to examine which anal behaviors other than receptive anal sex were associated with testing positive for rectal CT. Participants were provided with verbal and written instructions on how to self-collect rectal specimens, which is the clinic's standard-of-care. Self-collected rectal swabs were used as follows: one for culture (collected first) and one for NAAT (collected second). Participants immediately placed the culture swab into refrigerated 2SP transport media. The NAAT specimen collection kit and test used was the Aptima Combo-2 (Hologic, Inc., Marlborough, MA). NAAT specimens were tested within 5 business days; participants who tested positive for rectal CT were informed of the results and provided treatment. The culture specimen was frozen at −80°C until analysis. There were a total of 32 specimens positive for rectal CT by NAAT in this study.

### 2.4. Viability PCR

Samples were split into 2 equal volumes. One was treated with the gram-negative enhancer as recommended per Biotium protocol, and the PMAxx dye at 50 µm final concentration, according to manufacturer's instructions (Biotium Inc). Viability samples were incubated for 10 min in the dark at room temperature, then exposed to blue (465–475 nm wavelength) LED light for 20 min using the Glo-Plate Blue illuminator (Biotium Inc). The second volume was used for detecting total DNA. The total DNA samples were diluted to the same volume as vPCR samples with phosphate buffered saline and stored at room temp during the vPCR treatment.

### 2.5. DNA extraction

Genomic DNA from samples was extracted from untreated or PMAxx-treated samples using the PureLink gDNA extraction kit (Thermofisher). Samples were incubated with an equal volume of digestion buffer and 24 µl of proteinaise K and incubated at 55° for 1 h. Then we added 24 µl of RNAse, 120 µl of lysis buffer, and 120 µl of pure ethanol and mixed. Samples were bound to PureLink gDNA columns and washed per manufacturer's recommendations. DNA was eluted in 50 µl of elution buffer.

### 2.6. Heat-treatment and assessment of vPCR

CT was heat-killed by treatment at 95% for 15 min in a thermomixer. An untreated (live) aliquot from the same tube was mixed with heat killed bacteria at ratios of 100:0, 90:10, 70:30, 50:50, 30:70, 10:90, and 0:100. Samples were split into two equal aliquots and treated with PMAxx dye or not, as described above.

### 2.7. qPCR and digital PCR

Quantitative PCR for CT genome copies was done using a primer/probe set for the single copy outer membrane protein (*ompA*) gene of CT. This primer/probe sequence that detects all CT serovars was developed and validated by Jalal et al. (16). Forward primer: 5′-GACTTTGTTTTCGACCGTGTT-3′; reverse primer′-ACARAATACATCAAARCGATCCCA-3′; probe, 5′-ATGTTTACVAAYGCYGCTT-3′. We independently tested optimal primer/probe concentrations and annealing temperature and used optimum concentrations of 500 nm of each primer and 600 nm probe, with an annealing and extension temperature of 56°. Primers and probe were then mixed at a 20x concentration for use in PCR assays.

Quantitative PCR was carried out on a QuantStudio 5 instrument (ThermoFisher Scientific) with 20 µl final reaction volumes consisting of 10 µl of 2x PrimeTime gene expression master mix (IDT DNA), 1 µl of primer probe mix, 5 µl of eluted CT DNA, and 4 µl H_2_O. All samples were run in duplicate, qPCR was run for 40 cycles, and results were averaged over the duplicates.

For digital PCR, all samples were subjected to quantitative PCR in duplicate prior to digital PCR to verify that the input volume into digital PCR would be in range for accurate detection of positive copy numbers. Digital PCR was run on a Stilla Naica system according to manufacturer's recommendations, with 45 total cycle and a 56° annealing/extension temperature. Digital PCR reactions were 25 µl final volume and consisted of 5 µl of 5x ToughMix PCR Master Mix (Quantabio), 1.25 µl primer/probe mix, 2.5 µl fluorescein (passive reference dye for dPCR), 11.25 µl H_2_O, and 5 µl of DNA template. Digital PCR readouts of copy number per µl was converted to copy number per ml of input sample using the instrument software.

### 2.8. Statistics

Statistics were calculated using GraphPad Prism version 9.5.1.

## 3. Results

### 3.1. Comparing viability PCR to total DNA PCR in defined CT stock samples

**Figure 1:**
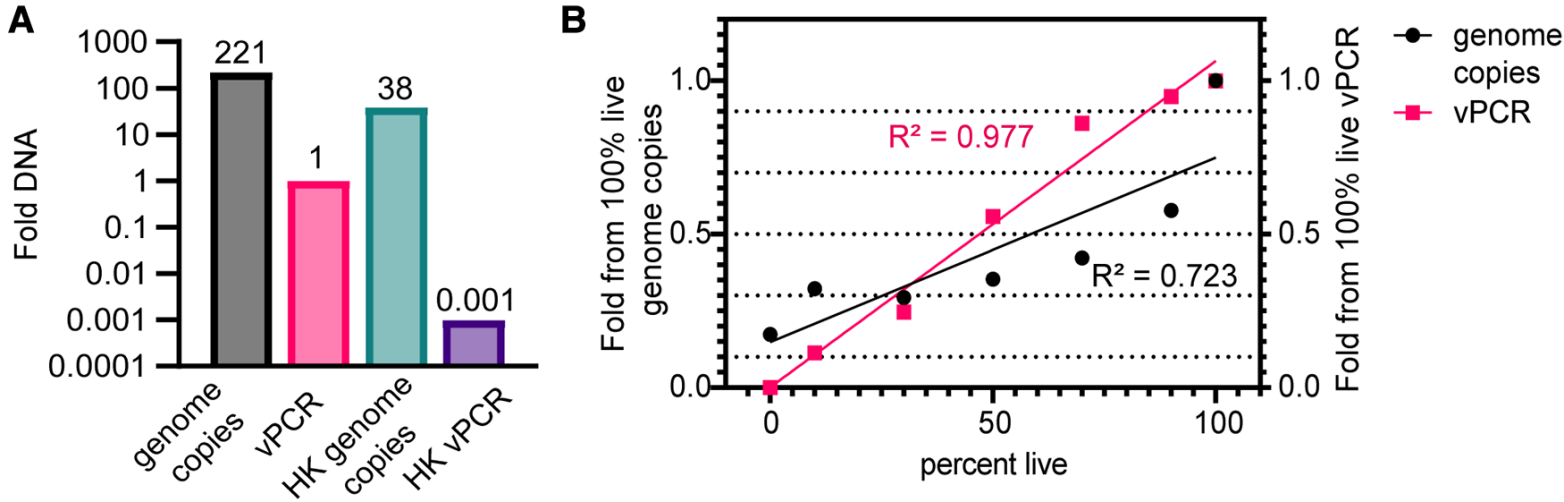
Technical validation of vPCR with heat-killed CT. Laboratory stocks of viable or heat-killed (HK) CT were subjected to quantitative PCR for total DNA or viable DNA following treatment with PMAxx reagent. PCR primers were designed to amplify the single copy *ompA* gene. (**A**) Total DNA and viable DNA results from freshly thawed and HK stocks. Bars are fold DNA copies relative to vPCR for freshly thawed stocks. The numbers show fold DNA differences for each category. Results were averaged from two technical replicates. (**B**) Freshly thawed and killed CT were mixed at 100%, 90%, 70%, 50%, 30%,10% and 0% viable CT by volume. Samples were split for PMAxx treatment or total DNA analysis by qPCR. Symbols are the relative DNA amounts for each sample type with 100% viable or total DNA set at 1 (for each sample type) plotted against percent live CT in sample. Dotted lines on Y-axis represent predicted amounts of DNA based on input ratios for each sample. Fitted lines and *R*^2^-values were calculated by simple linear regression.

We first tested freshly thawed and heat-killed laboratory-grown CT stock samples to determine how well viability PCR treatment distinguished between viable and non-viable bacteria. Due to loss of viability during CT stock preparation and purification and the freeze-thaw process, we expect a larger amount of total DNA than viable DNA in stock vials. In freshly thawed laboratory stocks, we detected 221-fold more total DNA copies than viable copies (Figure 1A). When CT were heat-killed (HK), we observed a drop of only about 6-fold (17% reduction, Figure 1A) for total DNA detection, demonstrating that total DNA PCR robustly amplifies DNA from non-viable bacterial. In contrast, vPCR detection dropped more than a thousand-fold for heat-killed samples (>99.9% reduction, Figure 1A). Next, we mixed heat killed CT with freshly thawed viable samples at defined ratios, and subjected samples to total DNA PCR and vPCR. We found that vPCR tracked much more closely with the expected drop in detection (*R*^2^ = 0.977 for vPCR vs. 0.730 for total DNA) (Figure 1B). This demonstrates that vPCR returns a more accurate estimate of the amount of viable bacteria in a sample of known composition.

### 3.2. Comparing vPCR to culture results for vaginal swabs

**Figure 2:**
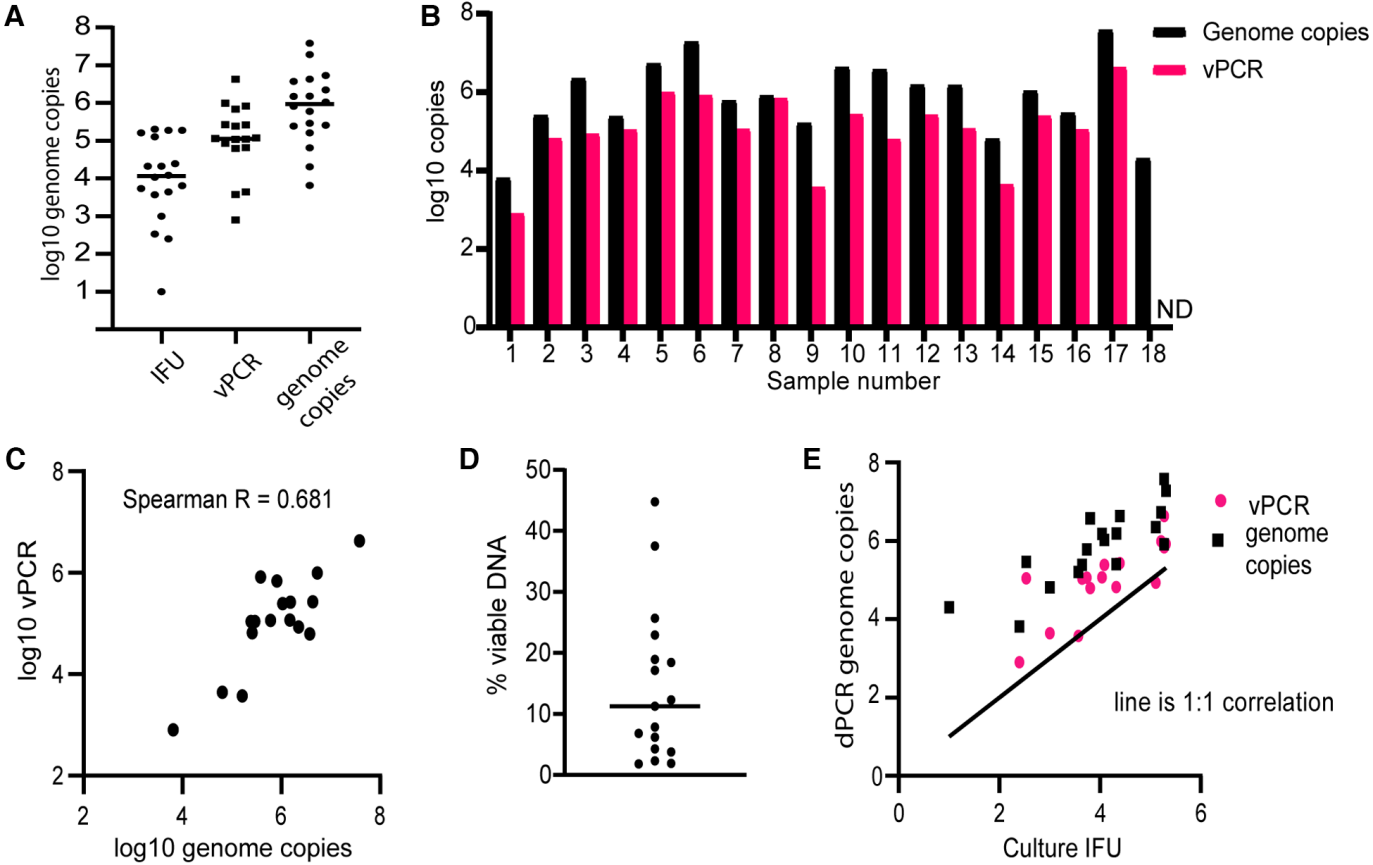
Comparing vPCR, total DNA, and culture results in vaginal swabs. Total DNA or viability reagent-treated DNA was extracted from vaginal swab samples and subjected to digital PCR amplification of the *ompA* gene. (**A**) Infection-forming units (IFUs) per ml and *omp*A copies detected per ml of sample volume in total and viability treated DNA. (**B**) Comparison of levels of total DNA and vPCR results for each sample. (**C**) Correlation between total DNA and vPCR results. (**D**) The percent of vDNA in total DNA copies for each sample. (**E**) Comparison of culture results with vPCR and total DNA results. The line is a hypothetical 1:1 correlation with culture results.

We used a set of 18 NAAT + clinical samples obtained by vaginal swabbing, all of which were also positive by culture assay. Culture results in samples ranged from 10 to 204,000 inclusion-forming units (IFU) per ml (Figure 2A, Supplementary Table S1). We used digital PCR (dPCR) to quantify the absolute number of viable and total *ompA* DNA copies per ml of sample. We detected a mean of 4,419,436 copies per ml of total DNA and 458,469 vPCR copies per ml in vaginal swabs (Figure 2A, Supplementary Table S1). For one sample which had only 1 IFU detected from 100 µl of input, we did not see any viable CT, but did detect 2,035 genome copies of total DNA. All other samples had more total DNA than viable DNA, and more viable DNA than culturable IFUs, as expected (Figures 2A,B).

Comparing the amounts of total DNA to viable DNA detected in these samples, we observed a moderate correlation (Spearman *R* = 0.681, Figure 2C). Viable DNA copies accounted for on average 14.35% of total DNA (range 1.81%–44.76%, Figure 2D).

Comparing dPCR results to culture results, vPCR detected between 0.667 and 323.53-fold more genome copies than culture (mean 28.37, standard deviation 76.46, median 9.77). Total DNA PCR detected far more genomes than IFU detected by culture, on average 884,223 fold more (standard deviation 1.8 × 10^6^ and median 2.6 × 10^5^). Both vPCR and total DNA strongly correlated with culture results (Figure 2E, Spearman correlation *R* = 0.843 for total DNA and 0.806 for vPCR), though vPCR estimates were closer to culture values in all cases, in Figure 2E, pink vPCR circles are closer to hypothetical 1:1 correlation line. Together, these results indicate that vPCR is a reasonably consistent estimate of viable bacteria. Only one sample detected less vDNA copies than culture IFUs and it was only 2.2% lower, indicating vPCR is not likely to miss culture-positive samples. While total DNA also correlated with culture results, it consistently overestimates the amount of viable bacteria in samples by a large factor.

### 3.3. vPCR results in rectal swabs

**Figure 3:**
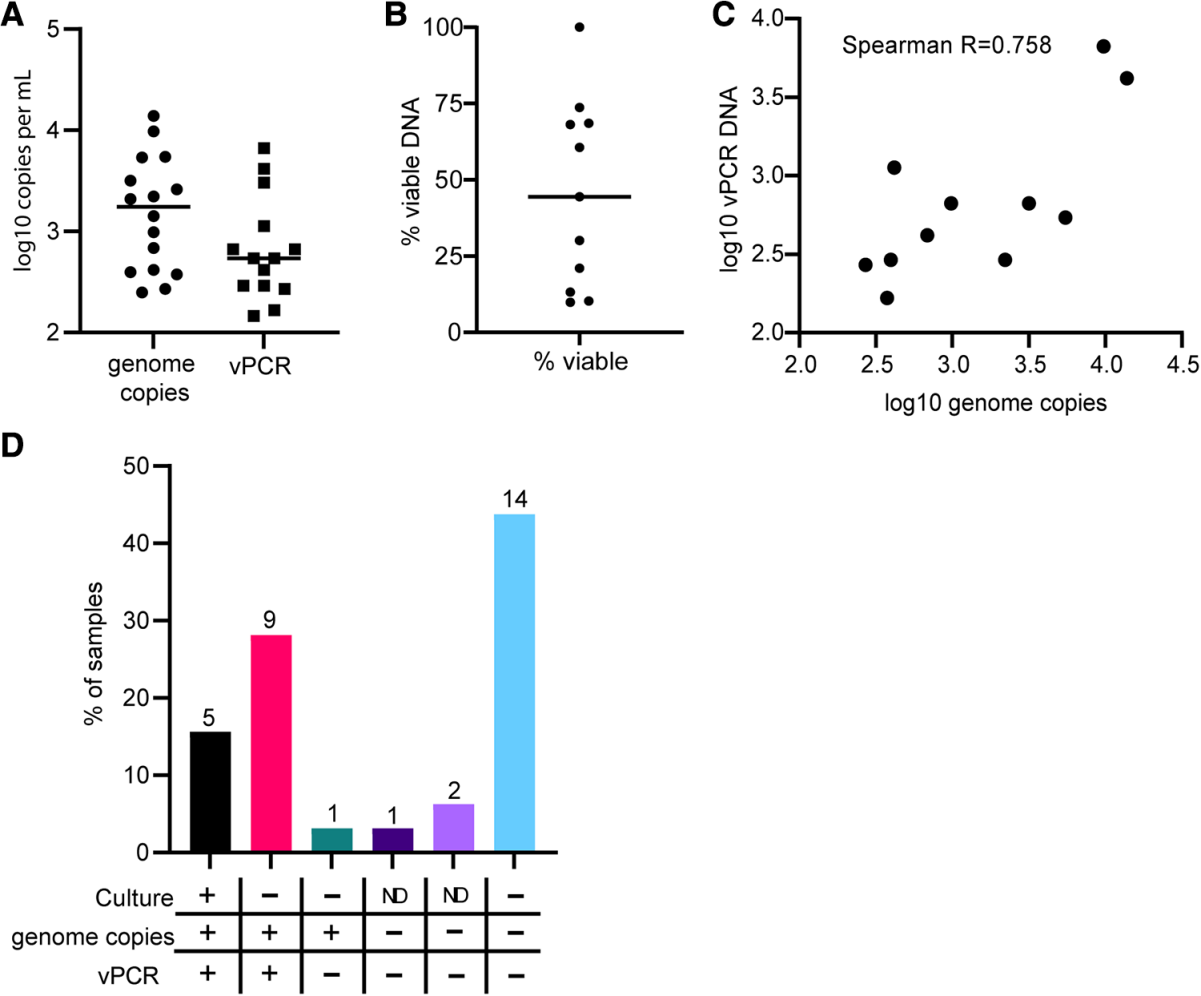
Comparing vPCR, total DNA, and culture results in rectal swabs. Total DNA or viability reagent-treated DNA was extracted from rectal swab specimens and subjected to digital PCR amplification of the *ompA* gene. (**A**) Comparison of levels of total DNA and vPCR results for each sample. (**B**) The percent of vDNA in total DNA copies for each sample. (**C**) Correlation between total DNA and vPCR results. (**D**) Percent of samples in each category of results. Numbers on top of bars are the total number of specimens in each category.

We also applied total DNA and vPCR analysis to 32 rectal swabs, all of which were positive for CT by NAAT assay. We attempted culturing on all swabs in two separate rounds, as well as conducting total DNA and vPCR amplification of *ompA* in all specimens. We report culture results as positive or negative because quantification of culture results is not reliable in rectal specimens (17). Out of 32 samples, we observed 5 positives by culture, 24 negatives by culture, and 3 were indeterminate in both culture attempts. In all 5 of the culture positive specimens we detected both total and viable DNA (Supplementary Table S2). We detected a mean of 3,081 copies per ml of total DNA, and a mean 1,357 copies per ml of viable DNA, which is more than 1,000-fold less total DNA and 338 fold less viable DNA than the mean for vaginal swabs (Figure 3A). In samples with both total and viable DNA, viable DNA accounted for 45.5% of total DNA on average (Figure 3B, range 9.9%–100%). Total DNA and vPCR copy numbers were strongly correlated in rectal swabs, as in vaginal swabs (Figure 3C). Of the 24 samples that were negative by culture, 14 did not have detectable CT total or viable DNA by PCR (Figure 3D). In 9 specimens that were culture negative, we detected viable CT DNA. In two specimens we detected only total DNA and no viable DNA copies (Figure 3D). Interestingly, in contrast to results in vaginal swabs, in three specimens (2, 29, 32) we detected more vPCR copies than total DNA copies. This suggests that in rectal samples there may be contaminating nucleic acids or PCR inhibitors that impair the detection of CT, which vPCR treatments prevent. Overall, 14 of the 32 (43.75%) of NAAT + rectal specimens had no detectable total DNA, viable DNA, and were negative by culture, 15.63% were positive by all 3 measures, and 40.63% were positive by only 1 method or indeterminate (Figure 3D). The concordance between negative culture results and negative PCR results suggests that there are NAAT + specimens that are unlikely to result from active rectal infections.

## 4. Discussion

This study is a validation of the vPCR method to exclude detection of non-viable *Chlamydia trachomatis*, as shown previously (9, 11). We found that vPCR is much less likely to detect dead organisms and correlates more closely with culture results than total DNA PCR. Our results suggest viability PCR can fill a gap in clinical CT research by assessing the likelihood that NAAT + results truly represent active infection.

Because we are interested in the application of this technique to clinical samples, we compared culture results, the current gold standard to detect viable CT, with total DNA and vPCR results on NAAT + vaginal and rectal swabs collected before treatment. NAAT assays are designed for maximum sensitivity and amplify nucleic acids that occur in numerous and variable copy numbers per CT bacterium. For example, 16s rRNA has been found to be 100–10,000 times more abundant by copy number than genomic DNA in trachoma (8, 18), and this number can vary by CT strain and metabolic state. Thus, NAAT results cannot be extrapolated to bacterial load measures. The *omp*A gene we are measuring occurs at only 1 copy per genome. Therefore, when using digital PCR, which returns absolute copy numbers, these results can theoretically be directly extrapolated to bacterial load. Furthermore, the large copy number per bacterium for NAAT amplicons could also result in NAAT positivity even in the absence of culturable CT or detectable viable DNA, due to lingering nucleic acids or detection of only dead or transient CT. This is of particular concern with NAAT + rectal samples.

In a set of 18 NAAT + vaginal swabs, one sample had only 1 IFU in culture, and we detected no viable DNA, though we did observe 20,350 total genome copies. This could represent a very early or almost resolved infection, or recent exposure without active infection. For all other samples we detected both viable and total DNA, both of which correlated with IFUs from culture. Overall, viable DNA accounted for 14.35% of the total DNA, but this varied from 1.81% to 44.76%. This demonstrates that the ratio of viable to non-viable DNA is not consistent in clinical samples. Likely during the course of an infection this ratio varies, starting with higher viable to total DNA ratios early or during the peak of infection, then, when immune responses start to control infection, more DNA from non-viable organisms is present. Or it could be a characteristic of different strains of CT with different intrinsic fitness. In only 2 specimens, vPCR was at or lower than culture results—the case with only 1 detectable IFU and for one specimen where vPCR detected 2% fewer total genomes than culture results. This suggests vPCR is unlikely to miss detection of culture positive samples, and provides a better estimate of the actual culturable amount of bacteria in a sample. Total genomic DNA copies is a vast overestimate of viable organisms, similar to NAAT results.

It has been hypothesized that CT inoculated orally could survive transit through the digestive tract and infect the lower GI tract (19, 20). However, it is also possible that oral inoculation with CT may instead just result in CT nucleic acids detectable in rectal swabs in the absence of active infection in the GI tract. Further, oral-anal sexual behaviors are very common practices among men who have sex with men (15, 21); these behaviors involve more superficial introduction of bacteria in the peri-anal region compared to insertive anal sex and thus rectal NAAT + specimens may be detecting just CT nucleic acid, not viable bacteria. Relying on NAAT positivity alone, in particular in rectal swabs, could potentially be resulting in treatments that are not necessary, in individuals with no actively replicating CT. Thus, an assay that can discriminate viable infection from the presence of CT nucleic acids, without the need for difficult culturing approaches is of particular importance as antimicrobial stewardship practices are needed in the clinical management of sexually transmitted infection (22–24).

Cultures of CT from rectal swabs are particularly difficult due to the high amount of contaminating bacteria, preventing quantification (we report results as positive or negative only), and making a large fraction of rectal culture results uninterpretable. Our rectal swabs came from a study population which reported no receptive anal sex. Although we anticipated observing some CT NAAT + rectal specimens, the total number we observed (*n* = 32) was nearly twice what we were expecting and was somewhat of a surprise. Regardless of the route of transmission, of 32 CT NAAT + rectal specimens from MSM enrolled in “Bottom's Up”, our vPCR failed to detect viable bacteria in 56% of the samples. These results are very similar to a recent study in women (10), which reported 48% of rectal swabs had no viable CT DNA. The detection of samples with likely active infection (positive by all three methods) and the detection of likely negative samples (negative by vPCR and culture) suggests that NAAT testing in rectal swabs picks up both active infection and transient or lingering DNA contamination which does not suggest a true infection. Viability PCR has the capacity to distinguish between these scenarios and is biased in the direction of over detection of DNA, therefore less likely to miss truly positive samples.

While this selective cohort may not be representative of all MSM undergoing rectal CT screening, the fact is that over half of CT-positive NAAT may be falsely positive—that is, not detecting true infection but errant DNA. In clinical practice, without the availability of vPCR, this could mean that half of all patients are receiving unnecessary antibiotics which may have long-term sequelae such as microbiome perturbations on the individual level (25), and antimicrobial resistance on the population level (22, 23). This current study alone does not provide enough evidence to change the current standard of care of offering antibiotic treatment to all NAAT + individuals given the risks of untreated Chlamydia infection, nor are we suggesting such a change be made at this time without such evidence. Future studies with frequent sampling of NAAT + but vPCR negative individuals could determine whether active infections ever arise in these cases, and only then inform clinical decisions about the pros and cons of antibiotic treatment.

Interestingly, in rectal swabs, in 3 cases we detected more viable DNA than total DNA, which was unexpected as total DNA should outnumber viable copies. This could be due to increased sensitivity of CT PCR following viability treatment with PMAxx dye. PMAxx dye is membrane impermeant, and enters dead microbes which have lost membrane integrity and binds to DNA. Following photolysis, the PMAxx-linked DNA has decreased solubility and is much less recoverable during DNA extraction. In rectal samples with high amounts of dead commensal bacteria, DNA recovery after PMA treatment is likely to be very different than total DNA recovery which includes DNA from all the dead commensal bacteria. The higher representation of non-CT DNA in total DNA samples could include PCR inhibitors or otherwise impair detection of total DNA relative to viable DNA. This could also explain why in rectal samples with both viable and total DNA, we detected a higher percentage of viable DNA per total DNA genome (45.5% for rectal samples vs. 14.35% for vaginal samples). This suggests digital total DNA PCR for *ompA* may not be a reliable measure of bacterial load.

The mechanism of dead DNA exclusion by PMA, which relies on microbe membrane integrity, could also result in overestimates of viable infection if bacteria killed by the immune system or are otherwise non-viable maintain membrane integrity. However, as the main hurdle to applying vPCR to clinical samples is worry about missing true positives, bias in the direction of over-estimation of true infection by vPCR is less of a concern.

Our study has relatively small numbers, but the very similar results to other studies of vPCR in CT + samples (9–12) suggests this is a reproducible and consistent approach to assessing true infection in clinical samples. Our Bottom's up study was focused on men who do not report receptive anal sex, which may bias our rectal samples towards those less likely to have true rectal CT infection, though these men did all have NAAT + rectal specimens. A cohort with more broad sexual practices might demonstrate a higher percentage of vPCR positive and culture positive swabs. We would still expect to see a fraction of rectal swabs with no evidence of active infection, which can easily be detected by vPCR. Future studies will investigate the rate of vPCR positivity in rectal swabs in expanded cohorts of participants.

In conclusion, we found that vPCR represents a reasonable, fast, and easy method to assess viability in clinical samples and correlates with culture results better than total DNA PCR. For any lab equipped to do PCR analysis of clinical samples stored in a manner amenable for culture, the process of viability treatment requires only one additional step (treating samples with PMAxx dye) and one additional piece of equipment (a photolysis device to cross link PMAxx to DNA). This process could easily be commercialized, clinically validated per CLIA regulations and scaled for clinical use. With increased movement toward antimicrobial stewardship in STI clinical practice, viability assays are an important tool that could reduce unnecessary antibiotic use.

## Data Availability

The original contributions presented in the study are included in the article/**Supplementary Material**, further inquiries can be directed to the corresponding author.
