# Supplementary material for: Use of viability PCR for detection of live Chlamydia trachomatis in clinical specimens
Source: Front Reprod Health. 2023 Aug 4;5:1199740. doi: 10.3389/frph.2023.1199740 (PMC10436598; doi:10.3389/frph.2023.1199740)
Supplement: Supplementary file 1 [file Datasheet1.pdf]

sample	IFU/mL	total DNA copies per mL	viable DNA copies per mL
1	250	6500	800
2	21000	257000	66000
3	127500	2242500	85000
4	4400	243500	109000
5	163000	5360000	987500
6	204000	19125000	831000
7	5440	604500	114500
8	188000	818000	692500
9	3740	161500	3750
10	24600	4330500	268500
11	6400	3770500	62500
12	21250	1531000	262500
13	10880	1502000	118000
14	1000	64500	4400
15	12240	1069500	245500
16	340	293000	110000
17	188000	38150000	4291000
18	10	20350	0

Supplemental Table 1: Culture, digital total DNA, and digital vPCR results for vaginal swabs. Vaginal swabs were thawed, diluted and cultured to calculate inclusion-forming units as described in the materials and methods. Samples for PCR were split, one was viability treated, then both viability treated and total DNA was assayed for *ompA* by digital PCR. Copies per mL of original sample are reported.

PTID	Culture outcome	Viability PCR	Concordance
1	Negative	ND	NEGATIVE
2	Negative	t = 417, v = 1125	vPCR+
3	Negative	ND	NEGATIVE
4	Negative	ND	NEGATIVE
5	Negative	t = 375, v = 167	vPCR+
6	Negative	t = 688, v = 417	vPCR+
7	Positive	t = 13,833, v = 4167	POSITIVE
8	Positive	t = 979, V = 667	POSITIVE
9	Negative	t = 3167, v = 667	vPCR+
10	Positive	t = 9729, v = 6667	POSITIVE
11	Positive	t = 5479, v = 542	POSITIVE
12	Contaminated	ND	ND
13	Negative	ND	NEGATIVE
14	Contaminated	t = 2604, v = ND	ND
15	Negative	t = 396, v = 292	vPCR+
16	Negative	t = 271, v = 271	vPCR +
17	Negative	ND	NEGATIVE
18	Negative	t = 2208, v = 292	vPCR+
19	Negative	t = 5396	total DNA+ only
20	Negative	ND	NEGATIVE
21	Negative	ND	NEGATIVE
22	Negative	ND	NEGATIVE
23	Negative	ND	NEGATIVE
24	Negative	ND	NEGATIVE
25	Negative	ND	NEGATIVE
26	Negative	ND	NEGATIVE
27	Contaminated	ND	ND
28	Negative	ND	NEGATIVE
29	Negative	t = 250, v = 541	vPCR+
30	Negative	t = 1417, v = 146	vPCR+
31	Negative	ND	NEGATIVE
32	Positive	t = 2083, v = 3042	Positive

Supplemental Table 2: Culture and PCR results from rectal swabs. Rectal swabs were tested twice in culture assay, results are from either one or both tests. Samples for PCR were split, one was viability treated, then both viability treated and total DNA was assayed for ompA by digital PCR. Copies per mL of original sample are reported T = total DNA, v = viable DNA, ND = not detected. Last column “Concordance” reports agreement between culture and PCR results, ND = not determined
